# The acceptability of the Fire and Rescue Service working with primary care to improve identification of mental health problems in older adults. A mixed-method qualitative study

**DOI:** 10.3399/BJGPO.2023.0059

**Published:** 2023-11-29

**Authors:** Tamsin Fisher, Carolyn A Chew-Graham, Nadia Corp, Saeed Farooq, Paul Kingston, Ian Read, Jane Southam, Gary Spolander, Dean Stevens, Mark Walchester, Carmel Warren, Tom Kingstone

**Affiliations:** 1 School of Medicine, Keele University, Keele, UK; 2 Midlands Partnership NHS Foundation Trust, Stafford, UK; 3 Centre for Ageing Studies, University of Chester, Chester, UK; 4 Staffordshire Fire and Rescue Service, London, UK; 5 School for Applied Social Studies, Robert Gordon University, Aberdeen, UK

**Keywords:** aged, anxiety, depression, Fire and Rescue Services, firefighters, non-traditional providers, older adults, primary care, referral and consultation

## Abstract

**Background:**

Mental ill-health in older adults (aged 60 years and over) is often underdiagnosed and undertreated. Older adults are less likely to access mental health services owing to perceived stigma and fear of being a burden. Non-traditional providers of health care, such as the Fire and Rescue Services (FRS), provide a possible solution to facilitate early detection of problems and help-seeking among older adults, especially in the context of pressured statutory services.

**Aim:**

To examine whether and how FRS Home Fire Safety Visits (HFSV) could be optimised to include detection and signposting for mental health problems — particularly anxiety and depression — in older adults.

**Design & setting:**

This mixed-method qualitative study took place in the West Midlands, UK in 2022.

**Method:**

This study involved focus groups (*n* = 24) and interviews with FRS staff (*n* = 4) to develop an in-depth contextual understanding of he acceptability and feasibility of expanding HFSV to include identification of anxiety and depression.

**Results:**

FRS staff were open to expanding their HFSVs to include mental health, provided they had sufficient training and support from partner agencies in primary and social care settings to accept referrals for service users presenting with symptoms of anxiety and/or depression.

**Conclusion:**

The positive reputation of FRS staff and engagement with older adults suggests that HFSV could support the detection of anxiety and depression in older adults, and appropriate signposting to other services including primary care.

## How this fits in

Older people are less likely to seek help for mental health problems, such as anxiety and depression. The Fire and Rescue Service (FRS) has existing points of contact with older people, living in their homes, through the provision of home fire safety visits. The FRS is an under-utilised healthcare asset which could support older people to access support for anxiety and depression, but this has not been explored previously. This research generated qualitative evidence, from the perspectives of FRS staff, on the acceptability of the FRS detecting mental health problems in older people and sign-posting to sources of support, such as, primary care.

## Introduction

The UK has an ageing population. In 2022, there were almost 11 million people aged 65 and over in the UK; this is expected to increase to 13 million by 2032.^1^ Symptoms of depression and anxiety are commonly experienced by older adults, often as a consequence of major life changes such as loss of role (for example, employment), bereavement, or chronic illness.^2,3^ One in four older adults (aged ≥60 years) experience symptoms of mental ill-health; however, just 15% of older people with a mental health condition are receiving care through the NHS.^4^ A variety of barriers can either delay or prevent help-seeking for anxiety and depression, including limited medical literacy around mental health, perceived cost, mobility and access, fear of being a burden,^4^ and stigma.^5^

Of those who accessed Improving Access to Psychological Therapies (IAPT) services in 2014/15, only 7% were older adults.^2,6^ Referrals for older adults into IAPT services is low at 3.5%, and people aged >85 years are five times less likely to be referred to IAPT services than younger people.^7^ Left untreated, anxiety and depression can worsen outcomes of comorbid physical health problems^8^ and increase mortality.^9^ Therefore, early access to health services provides an opportunity for appropriate intervention and enhanced long-term outcomes.^10^

Health and social care services in the UK are currently overwhelmed by demand.^11^ FRS has a history of working with health and social care on interventions such as fall prevention,^12,13^ dementia awareness,^14,15^ and as emergency responders to cardiac arrests.^16^ The NHS has a policy to engage non-traditional providers of health care^17^ to help detect health problems. FRS has been identified as a key asset through publication of a consensus statement between NHS England, Public Health England, and the Chief Fire Officers’ Association.^18–20^

Throughout the COVID-19 pandemic, the FRS was utilised to bolster health and social care services by driving emergency response vehicles, assisting with patient care under the supervision of ambulance clinicians,^21^ and supporting the national COVID-19 vaccination programme, including by administering vaccines. Thus, the FRS is adopting important roles in health and social care. They are able to do this through being embedded in their local communities and having the privilege of public support and trust.^22^ However, working beyond the normal boundaries of the job can present challenges in terms of role expectations and perceptions, both among the public and within the FRS.^18^

This article reports qualitative research conducted as part of a mixed-method study (the FIRESIDE study), which explores whether and how existing Home Fire Safety Visits ([HFSV], visits made by the FRS to members of the public identified as being at risk of home fires) could be adapted to identify anxiety and depression in older adults and provide initial signposting. Key risk factors that determine who receives an HFSV include age and frailty. This point of contact between older adults and the FRS could be a useful opportunity to raise awareness about anxiety and depression. Age UK have recommended that health and social care staff working with older adults should receive awareness training to help identify and respond to signs and symptoms of mental health problems.^4^ However, there is an evidence gap around the potential role of the FRS in delivering mental-health-related interventions.^18^ In this qualitative study, the views of FRS staff about experiences of delivering HFSV to older adults were explored, expanding their HFSV to include mental health awareness, and what FRS staff might need, in terms of training, to deliver an expanded HFSV.

## Method

### Research design and setting

A qualitative methodology was used to explore the subjective experiences and opinions of FRS staff, in line with the aim of the study. Qualitative methods included semi-structured interviews and focus groups.

### Recruitment

Recruitment targeted FRS staff with experience of delivering HFSV; a purposive approach to sampling was applied to ensure diversity in terms of sex, role type, role location, and time in service. All participants were recruited via existing contacts at one FRS in the West Midlands, England. This service covers a richly diverse county in terms of levels of deprivation, and demography, urbanicity, and rurality. Three different staff groups were identified as responsible for delivering HFSV:

- technicians (who deliver HFSV as part of community outreach, instal fire safety equipment, and discuss fire safety in people’s homes; any needs beyond this are referred to community safety officers [CSOs] rather than to external agencies);
- CSOs (who deliver HFSV as technicians do, but have capacity to make referrals to partner agencies); and
- operational crews (who are required to complete HFSV in addition to their primary role as firefighters).

All data collection took place between February and November 2022. The eligibility criteria for participation were: (*a*) an employee for FRS, (*b*) experience of conducting HFSV, and (*c*) the capacity to consent to participation in a focus group or individual interview.

### Data collection

In-depth semi-structured interviews were conducted either face-to-face or via an online platform. Focus groups were conducted on FRS premises to provide accessible opportunities and encourage participation. Key demographic information was collected at the start of the focus group (including age, sex, job role, location, and time in service).

Topic guides were used for both interviews and focus groups (available as a supplementary document). The topic guides were modified as data collection and analysis progressed. Data were collected until data saturation was reached and it was felt that no new depth of information arose from the focus group.

All interviews and focus groups were recorded using a digital audio-recording device. Recordings were transcribed by a professional transcribing service (www.thetranscription.co.uk); transcriptions were anonymised by TF before analysis. All personal information, including names, was removed, and each participant was assigned a pseudonym using the format FRS[n].

### Analysis

All transcripts were analysed using a thematic approach^23^ with principles of constant comparison. Regular analysis meetings took place between three authors (TF, TK, and CC-G) and preliminary themes were discussed and finalised with the wider multidisciplinary research team and patient and public involvement and engagement (PPIE) group members.

### Patient and public involvement and engagement

A PPIE group of six adults with lived experiences of mental ill-health (personal and/or as a caregiver) met with the research team throughout the study, and contributed to the methodological approaches and the design of topic guides.

## Results

Participants varied in job role (technicians [*n* = 9], CSOs [*n* = 10], operational crew members [*n* = 5], and trainers/managers [*n* = 4])(Table 1); and in age (mean = 43.6, range 20–63 years), sex (female = 32.1%, male = 67.9%), and time in current role (7 months to 14 years)(Table 2). The mean time for focus groups was 97.3 minutes; the mean time for interviews was 55.3 minutes.

**Table 1.** Job role of participating FRS staff

Job role	*N*	Data acquisition method
Technicians	9	Focus group
CSOs	10	Focus group
Operational crew members	5	Focus group
Managers/trainers (one-to-one interview only)	4	One-to-one interview

CSO = community safety officer. FRS = Fire and Rescue Services.

**Table 2.** Demographics of participating FRS staff

	Technicians, *n*	CSOs, *n*	Operational crew, *n*	Mean age, years	Sex (male), *n*	Mean length of service, years
Focus group 1	8	0	0	50.9	8	8.1
Focus group 2	0	6	0	31.7	2	2.4
Focus group 3	0	0	5	38.4	3	8.9
Focus group 4	1	4	0	52.4	4	5.8
Interviews	Managers/trainers of technicians/CSOs			42.7	1	8.0

CSO = community safety officer. FRS = Fire and Rescue Services.

Four key themes will be presented here: a foot in the door; witnessing distress in older adults; operating on the outside of health and social care; and scoping out a role in mental health. Data extracts are included and identified by a unique participant code (FRS[n]).

### A foot in the door

Participants described their unique position in the community compared with other agencies, including health and social care providers. FRS staff are generally perceived as trustworthy and able to get a ‘foot in the door’ where other services may struggle:

‘*The badge definitely helps to get us in which is why some services ask us to go in because they think, “You’re going to get in*”.’ (FRS09)

Most of the technicians and CSOs commented that their job required them to be good at building rapport and developing positive relationships with service users. The FRS staff recognised the respect that they receive from their communities, but also commented that respect is reciprocated:

‘*We’ve got a really good sort of rapport and we’ve got a really good bond with the members of the public … we’ve got a really strong presence and I think we’re well respected, but I think we respect them so much as well, don’t we?*’ (FRS06)

FRS staff suggested that while service users generally welcome the HFSV, there were exceptions, and staff reported that some people may not be aware that FRS support the community outside of preventing fires:

‘*I’ve had a couple of clients recently where they’re like, “Well you’re from the fire service, what can you do for me?” And it’s hard getting across that we do a lot more than just fitting a smoke detector*.’ (FRS23)

### Witnessing distress in older adults

Participants described examples of visiting people with mental ill-health including anxiety, depression, hoarding disorder, dementia, and suicidal ideation.

CSOs described being exposed to highly vulnerable service users with underlying mental health problems, and referred to visits with older adults living chaotic lifestyles. Participants shared examples of visits that they had found personally distressing:

‘*He* [service user] *was saying he wanted to die. He said he wasn’t going to eat and he wasn’t going to turn his heating on. He was living on a farm in the middle of nowhere. If he died that day, it would have been weeks before he was discovered…*’ (FRS13)

FRS staff commented that they regularly work with service users who they suspect might have mental health problems, but they could not be certain:

‘*Sometimes you know there’s something wrong with the person, but you can’t put your finger on it can you? So they’ll answer the question sometimes, but you can just tell that something is perhaps not quite right*.’ (FRS27)

Some FRS staff commented that they see *‘some terrible stuff*’ (FRS25) and are exposed to distressing situations in their job; however, they lack the necessary training and awareness to know how to deal with these situations:

‘*I’ve had a few incidents where mental health took over. I don’t know how to deal with it ... I don’t have a clue what to say or know whatever is the right thing to say. How are you supposed to identify what the right thing to say is? We’re not trained on that and we’re not specialists. We go in and ask the questions and I actually think that one of our questions in our Home Fire Safety Visits is “Are you depressed?” But if they say yes, what do I say?*’ (FRS11)

Exposure to situations involving people with suspected or confirmed mental ill-health either at low or high levels was reported to have a detrimental effect on staff wellbeing:

‘*You do find you take it home, especially when I had a safeguarding denied*.’ (FRS13)

### Operating on the outside of health and social care

As part of the HFSV, FRS staff said they routinely ask questions about mental health (*Are you depressed*), loneliness (*Are you lonely?*), and medication use (*You don’t have to tell me what it is, but do you take any medication that may hinder your ability to get out of the property in an emergency; for example, medication that makes you drowsy?*). However, despite covering topics related to health and social care during home visits, FRS described various ways in which they operated on the outside of health and social care, owing to a lack of awareness of services and referral systems. Relationships between FRS and health and social care partners were generally described as positive but challenges with the referral process exist:

‘*When I work with somebody, they are very good. But trying to get somebody into that system sometimes is damn near impossible*.’ (FRS26)

Participants also raised concerns about barriers to making successful referrals into health and social care services. Thresholds were a common source of frustration:

‘*A thing we often hear is “it doesn’t meet the threshold” and “has* [cognitive] *capacity”*.’ (FRS09)

Referral pathways into services were described as unclear by participants. Referrals into mental health services were described as particularly problematic:

‘[*…*] *I think if we’re asking the questions, we need an adequate referral pathway to follow that up. So we’re not just asking questions, storing it and not doing anything about it. We need a route to help that person*.’ (FRS16)

This frustration can lead to a sense of hopelessness among FRS staff:

‘*You don’t feel like you’re really helping because there’s nothing you can do.’* (FRS10)

Participants described variation in their relationship with primary care services, specifically local GPs. The FRS does not routinely record the GP data of the service users who they visit; participants described challenges accessing to primary care services:

‘*Especially with GP appointments, we’re currently struggling. I’m sure everyone has had the problem of trying to get hold of the doctors and being in a line of phone calls*.’ (FRS11)

One participant however, described experiencing a proactive response from a GP:

‘*The one I went to … they were quite worried and sent a message directly to the GP and the GP went out to see him … it depends on the GP really, on the practice and how they work.*’ (FRS25)

Another participant described attending a GP surgery in person to speak directly with the receptionist:

‘*If it’s the community and you’re driving past. I’ll call in and face to face is always better than a telephone call.*’ (FRS26)

### Scoping out a role in mental health

Most participants were open to receiving additional mental health training:

‘*I think people would want to feel trained and that they’ve got the adequate knowledge to be able to know where to refer as well. We can ask questions, but know that we’ve got support from partners that we can refer to and that will kind of assist and take that referral on for us…*’ (FRS16)

One participant specified topics they felt would be useful in their everyday job:

‘*I’d like to first of all help spot these signs and symptoms and also how to then expand that conversation of what can we offer?*’ (FRS19)

However, in terms of the potential level of support and engagement that they could provide to members of the public in the context of mental health, participants expressed concerns about the scope of their role and the need to maintain clear boundaries with other professions:

‘*You can get too involved if you’re not careful on one specialised subject. I mean, we’re not mental health nurses or consultants or doctors, we are firefighters, fire service, whatever way you want to put it*.’ (FRS02)

Participants shared concerns about capacity within the health and social care system; this reinforced a sense of hopelessness in identifying concerns in the first instance, and then in attempting to make referrals:

‘[…] *it’s establishing as well that if we’re going to go in and ask more questions around mental health … where is the additional support out there … people are getting appointments in two weeks’ time and they’re banging their head off a wall. If you’re going to go into that in more detail, it’s doing that with the support of somebody who can actually pick up on that then, because otherwise, what’s the point?*’ (FRS13)

However, some FRS staff were sceptical that this would be possible when they are limited on time to conduct a HFSV:

‘*It’s still going to be hard to know how much training you have. You cannot spot the signs and symptoms between us five and spend twelve hours a day with someone.*’ (FRS21)

Participants shared a willingness to engage with more mental health training; however, concerns were raised about role boundaries, the extent and scope of training, and the feasibility of what can be achieved during an HFSV. Broader issues around capacity in the system and referral pathways were also described.

## Discussion

### Summary

The findings of this study help to illuminate aspects of public trust in the FRS, exposure of FRS staff to mental health among members of the public, a sense of the FRS acting as an outsider to traditional health and social care providers, and the potential scope of a new intervention or role.

It seems evident that FRS staff witness distress among members of the public, including older adults, and therefore have an opportunity to offer support. Participants acknowledged the high level of trust that members of the public afforded them, which enabled them to get a foot in the door where other services were not permitted the same level of access. Participants recognised that they could support partners in primary care to identify mental ill-health in older adults; however, barriers were identified.

FRS staff do not currently feel that they have the necessary support from partner agencies, and often find that referrals that they make do not meet the threshold required for care. FRS staff also admitted to contacting GPs on behalf of service users to arrange appointments. A lack of familiarity or awareness with health and social care systems and referral processes, including thresholds, were commonly described, often as a source of frustration. Several concerns were raised about the scope of a potential mental health role, much of which centred upon feasibility and traditional role boundaries. Despite these concerns, participants expressed a willingness to engage in further mental health training to feel better equipped on HFSV.

### Strengths and limitations

This novel study helps to address the evidence gap around the potential role of the FRS in supporting older adults, in the context of mental health conditions such as anxiety and depression. The research team worked in partnership with FRS managers. The FIRESIDE study was informed by PPIE contributors throughout.

The sample included a diverse range of participants in terms of sex, age, job role, time in role, and locality. The multidisciplinary research team increased the trustworthiness of the data analysis. Participant recruitment took place in a single service. Findings may not reflect the views of staff in other services and localities. All participants were White British; however, this reflects the workforce within the service.

### Comparison with existing literature

Existing literature on the role of FRS in the identification of mental health problems is scant,^18^ especially anxiety and depression. Previous studies have explored the challenges of implementing an intervention in the FRS, and FRS staff’s perspectives of expanding their role to support the ambulance service and conduct HFSV (also known as Safe and Well visits in some FRS).^16^ FRS staff felt that they had the clinical skills to support the ambulance service, but lacked the emotional skills. Byrne-Davis *et al* highlighted similar barriers to the Safe and Well visits more generally, including the need for additional training.^16^ Our study contributes to the literature more specifically, regarding the extension of HFSV to address mental ill-health among older adults. This study also enhances the current evidence base that there is a need for further training and development in mental health awareness within the FRS. Any training intervention produced would take inspiration from mental health first aid training, with particular attention being paid to anxiety and depression, while also considering the nature and delivery of the intervention based on findings from a recent realist review, RIDDLE.^18^ The development of an intervention for the FRS will be complex to ensure it fits within the existing system. Kingstone *et al* found that many similar interventions have taken a one-size-fits-all approach, which was considered problematic for this setting.^18^ Nationally, the FRS is transitioning its HFSVs to use a person-centred framework approach.^24^

FRS staff are trusted and respected by members of the public and health and social care stakeholders, as evidenced by the publication of numerous consensus statements between the Chief Fire Officers’ Association and partners, including NHS England.^4,20,25^ Although they are not traditional providers of care, the FRS has historically been employed by the NHS and primary care to support work in the community.^12,16^ The NHS Long Term Plan^17^ highlights the need to utilise services such as the FRS, who have shown success in short term support; however, the FIRESIDE study demonstrates potential for an extended, and even permanent, role of the FRS in supporting primary and social care providers to identify and refer and/or signpost to relevant services (provided the FRS staff receive sufficient training and support).

Integrated care must involve the establishment of referral pathways, which includes awareness of the eligibility criteria for service entry. This has been essential in similar intervention models,^20,26^ such as interventions utilising social prescribers. Social prescribers faced challenges similar to those of the FRS participants, around building relationships, communication techniques, and having a shared understanding of service provision with health and social care partners (for example, GPs);^27^ these concerns were also raised by participants.

### Implications for research and practice

This study will help to inform the design of a new intervention to be delivered by FRS during HFSV to support identification of, and signposting for, anxiety and depression in older adults. The current study raised some areas for further exploration, which will need to be addressed before any intervention can be designed. Concerns about health and social care systems and capacity were raised; it is therefore important to examine perspectives of relevant health and social care stakeholders, including those in primary care, about the potential role of the FRS in the detection of anxiety and depression in service users in their routine work. It is also crucial that the views of older adults, as intended recipients of any such intervention, be sufficiently explored and incorporated. The authors are currently addressing these evidence gaps with ongoing research.

The role of fire prevention teams in the fire service is to prevent incidents of fire, and FRS staff are not — nor should they be — mental health professionals. However, they could be better utilised to support health and social care partners to identify older adults presenting with anxiety and depression. The FRS is already working with people living with mental ill-health that surpasses their expertise and training, and FRS staff want to be better equipped to support them. There is an opportunity for primary care to utilise the widely respected reputation of the FRS, who have the privilege of access to properties many organisations do not. Most FRS staff felt that they could identify older adults living with anxiety and depression during the HFSV, provided that they have appropriate training and sufficient referral and/or signposting pathways. If the FRS do expand the HFSV, they need support from primary and social care partners such as GPs and mental health professionals to appropriately support the needs of service users. With the development of referral pathways, non-traditional providers of care such as the FRS could become important partners within integrated care systems. Training and improved understanding of these systems would help their ability to make informed decisions, and signpost service users to the appropriate care. Further opportunities for closer working and collaboration would be welcome.

The FRS is potentially well placed to support the detection of and signposting for mental health problems in older adults, owing to the access they have to older adults through their existing roles and the level of respect they hold among the public. The FRS do not currently have a standard reporting system to raise mental health concerns, and may lack the necessary awareness to successfully navigate health and social care systems to benefit their service users. To design any intervention in this area, several barriers need to be addressed, and adequate training needs to be developed and delivered to ensure FRS staff are competent and confident to approach mental health concerns among older adults. System-level challenges may be beyond the scope of any one intervention; however, having greater opportunities for FRS to work together with health and social care providers, including primary care, will enhance mutual awareness of the other’s role in the system, and support navigation and communication.

## Data

The dataset relied on in this article is available from the corresponding author on reasonable request.

## References

[1] Centre for Ageing Better (2022). The State of Ageing in England is getting worse. https://ageing-better.org.uk/summary-state-ageing-2022#:~:text=Today%20there%20are%20almost%2011%20million%20people%20aged%2065%20and%20over%20%2D%2019%25%20of%20the%20total%20population.%20In%2010%20years%E2%80%99%20time%2C%20this%20will%20have%20increased%20to%20almost%2013%20million%20people%20or%2022%25%20of%20the%20population.

[2] Laake J-P, Majeed N, Walters K (2021). Realising the potential of improving access to psychological therapies for older adults. Br J Gen Pract.

[3] Kingstone T, Burroughs H, Bartlam B (2017). Developing a community-based psycho-social intervention with older people and third sector workers for anxiety and depression: a qualitative study. BMC Fam Pract.

[4] Age UK (2019). Mental Health (England): Policy Position Paper. https://www.ageuk.org.uk/globalassets/age-uk/documents/policy-positions/health-and-wellbeing/ppp_mental_health_england.pdf#:~:text=15%25%20Proportion%20of%20older%20people%20with%20mental%20health,people%E2%80%99s%20mental%20health%20needs%20are%20not%20left%20behind.

[5] Chew-Graham C, Kovandžić M, Gask L (2012). Why may older people with depression not present to primary care? messages from secondary analysis of qualitative data. Health Soc Care Community.

[6] Pettit S, Qureshi A, Lee W (2017). Variation in referral and access to new psychological therapy services by age: an empirical quantitative study. Br J Gen Pract.

[7] Frost R, Beattie A, Bhanu C (2019). Management of depression and referral of older people to psychological therapies: a systematic review of qualitative studies. Br J Gen Pract.

[8] National Institute of Mental Health (2021). Chronic illness and mental health: recognizing and treating depression. https://www.nimh.nih.gov/sites/default/files/health/publications/chronic-illness-mental-health/recognizing-and-treating-depression.pdf.

[9] Meier SM, Mattheisen M, Mors O (2016). Increased mortality among people with anxiety disorders: total population study. Br J Psychiatry.

[10] Farrer L, Leach L, Griffiths KM (2008). Age differences in mental health literacy. BMC Public Health.

[11] Alderwick H (2022). Is the NHS overwhelmed?. BMJ.

[12] Lowton K, Laybourne AH, Whiting DG, Martin FC (2010). Can Fire and Rescue Services and the National Health Service work together to improve the safety and wellbeing of vulnerable older people? Design of a proof of concept study. BMC Health Serv Res.

[13] Laybourne AH, Martin FC, Whiting DG, Lowton K (2011). Could Fire and Rescue Services identify older people at risk of falls?. Prim Health Care Res Dev.

[14] Derbyshire Fire and Rescue Service Dementia. https://www.derbys-fire.gov.uk/community/health-and-wellbeing/mental-health/dementia.

[15] Heward M, Kelly F (2020). Research and education to understand fire risks associated with dementia: a collaborative case study (innovative practice). Dementia (London).

[16] Byrne-Davis LMT, Marchant D, Bull ER (2019). How do members of a fire and rescue service perceive expanding their roles to deliver more health care services?. J Public Health (Oxf).

[17] NHS England (2019). The NHS Long Term Plan. https://www.longtermplan.nhs.uk/wp-content/uploads/2019/08/nhs-long-term-plan-version-1.2.pdf.

[18] Kingstone T, Chew-Graham CA, Corp N (2022). Interventions to identify and manage depression delivered by 'nontraditional' providers to community-dwelling older adults: a realist review. Health Expect.

[19] National Institute for Health and Care Excellence (2016). Winter warmth pilot with Public Health England (PHE). https://www.nice.org.uk/sharedlearning/winter-warmth-pilot-with-public-health-england-phe#:~:text=The%20focus%20of%20the%20pilot,other%20services%2C%20including%20accident%20and.

[20] Public Health England (2016). Evaluation of the impact of fire and rescue service interventions in reducing the risk of harm to vulnerable groups of people from winter-related illnesses. https://assets.publishing.service.gov.uk/government/uploads/system/uploads/attachment_data/file/573558/FRS_winter_pressures_evaluation.pdf.

[21] National Fire Chiefs Council Covid 19: Ambulance assistance guidance. https://www.nationalfirechiefs.org.uk/write/MediaUploads/COVID-19/Tripartite%20agreements/NFCC_assisting_ambulance_guidance_FINAL.pdf.

[22] BMG Research (2018). Public perceptions of fire and rescue services in England 2018.

[23] Braun V, Clarke V (2019). Reflecting on reflexive thematic analysis. Qual Res Sport Exerc Health.

[24] National Fire Chiefs Council Person centred framework. https://www.ukfrs.com/person-centred-framework#:~:text=The%20NFCC%20Prevention%20Programme%20are%20pleased%20to%20announce,fatalities%20and%20serious%20injuries%20in%20the%20home%20setting.

[25] Association of Ambulance Chief Executives (AACE) and the Chief Fire Officers Association (CFOA) (2016). Consensus statement on saving lives and improving health and wellbeing. file:///C:/Users/ggb78/Downloads/CFOA-AACE%20consensus%20statement.pdf.

[26] Public Health Institute, Liverpool John Moores University (2019). An evaluation of the Fire & Rescue Service Safe & Well Visits in Cheshire and Merseyside.

[27] Pescheny JV, Pappas Y, Randhawa G (2018). Facilitators and barriers of implementing and delivering social prescribing services: a systematic review. BMC Health Serv Res.

